# Determinants for COVID-19 vaccination intention in Mexico

**DOI:** 10.1016/j.heliyon.2023.e18079

**Published:** 2023-07-18

**Authors:** Arcelia Toledo-López, Sandra Nelly Leyva-Hernández, Julio César Jiménez-Castañeda, María del Carmen Avendaño-Rito

**Affiliations:** a Instituto Politécnico Nacional, CIIDIR Oaxaca, Hornos 1003, 71230, Oaxaca, Mexico; b Universidad Autónoma de Baja California, Facultad de Ingeniería y Negocios San Quintín, Carretera Transpeninsular Km. 180.2, Ejido Padre Kino, 22930, San Quintín, Baja California, Mexico; c Instituto Tecnológico Del Valle de Etla, Abasolo S/N, Barrio Del Agua Buena, 68230, Oaxaca, Mexico

**Keywords:** Theory of planned behaviour, Model of goal-directed behaviour, Health belief model, Mexico

## Abstract

This investigation aims to determine the predictors that have the most significant influence over COVID-19 vaccination intention for the population of 18 years or above in Mexico. This will be done through a comprehensive theoretical model comprising: the theory of planned behaviour, the health belief model, and the model of goal-directed behaviour. An exploratory, cross-sectional study with a quantitative approach was carried out. The structured questionnaire was applied to 1085 adults in the first trimester of 2021 through Google Forms in social media groups. The data analysis was carried out through partial least square structural equation modelling. Positive anticipated emotions, desire, subjective norms, attitude, and perceived behavioural control were the most significant predictors of intention. The model that combines the theoretical perspectives explains mostly the vaccination intention. The study can be a valuable theoretical perspective for understanding similar behavioural intentions related to health risks. The results are also valuable for public health decision-makers to design strategies that promote vaccination.

## 1 Introduction

December 2019 was discovered a health emergency COVID-19 [1], changed the daily lives and activities of the population due to the rapid spread of the new coronavirus worldwide. To mitigate the spread of COVID-19, some governments have imposed measures such as social distancing, although their effectiveness depends on how strictly it is followed by the population [2]. As such, since the pandemic was declared, the most awaited product for the world's population was the COVID-19 vaccine.

In the first trimester of 2021, the world was experiencing an environment of uncertainty due to the number of COVID-19 cases and deaths and the emergency approval of vaccines to reduce infectious diseases [3,4]. In March of 2021, on a global scale, there were 113,820 confirmed cases, the majority of which were in America and Europe, and 2,527,891 deaths, with a worldwide mortality rate of 2.2% [3]. In December 2020, the World Health Organisation [4] emergency approved Pfizer-BioNTech, AstraZeneca and Janssen vaccines. Meanwhile, in Mexico, more vaccines were approved for use in an emergency: Pfizer-BioNTech, AstraZeneca, Sinovac, Cansino, and Gam-COVID-Vac [5]. The first vaccination for adults started on December 24 in 5 phases. Vaccination started with frontline healthcare workers of COVID-19 (phase 1). In Mexico, at the end of the first quarter, the vaccinated adult population was only 13.45%; when phase 2 of vaccination had finished, those in the middle sector and adults over 60 were vaccinated [6,7].

Even though various vaccine options exist, anti-vaccine attitudes and vaccination hesitancy have increased [8]. According to WHO [9], vaccination hesitancy halts the fight against diseases, for COVID-19. In a systematic review and meta-analysis of 38 articles about the acceptability of vaccination against COVID-19, Wang et al. [10] found that the factors that influence the acceptance or hesitancy of the COVID-19 vaccine were gender, age, educational level, household income, trust in the government, and history seasonal influenza vaccination, perceived risk, and beliefs on vaccines. Similar results were found by Wang et al. [11] in parental willingness to vaccinate children during the COVID-19 pandemic; they found that in addition to the socio-demographic characteristics of the parents, the attitude, the source of information of the immunization promoters, fear of needles and beliefs that vaccinations are unnecessary were the determinants of vaccination willingness.

The extensive literature on COVID-19 vaccination agrees that low educational level, age, low-income levels, ethnicity, gender, and occupation, as well as not having received similar vaccines, the perception of government measures as inadequate, a lack of trust in pharmaceutical companies, in health services, and the perception of information from the health authorities as inconsistent and contradictory, the beliefs on natural immunity, safety, and side effects of vaccines have been the main predictors for adults to be vaccine hesitancy or delay vaccination against COVID-19 [[10], [11], [12], [13], [14], [15]]. For example, studies conducted in different countries found that women, black people, adults. 60 years old with high school education, and those concerned about vaccine safety and side effects were the most hesitant about the COVID-19 vaccine [10]. Adults not vaccinated against seasonal influenza perceived lower risks and benefits of the COVID-19 vaccine and untrust scientists and public authorities involved in COVID-19 vaccines refusing COVID-19 vaccines [10,14].

This hesitancy is the greatest challenge that vaccine campaigns face [16]. It is present in various sectors of the population, even in those expected to have a greater vaccination intention. For example, among the population of healthcare workers (HCWs), 25% of health workers in Saudi Arabia have doubts about getting vaccinated against COVID-19 [8], 25.9% in France also have doubts about getting vaccinated [17]; 19.1% in Canada declined vaccination [12], and in Palestine, the 37.8% got vaccination intention, 31.5% were undecided, and 30.7% refused the COVID-19 vaccines [18]. Li et al. [16] identified that 18% of adults have doubts about getting vaccinated or do not want vaccinated in the eastern, western, and central regions of China, equally so in both rural and urban areas. They also found that adults above the age of 58 had the least disposition toward vaccination. In Italy, 15% of adults would decline the vaccine, and 26% doubt about having it administered [19]. In Portugal, vaccine hesitancy is elevated, given that 56% want to delay the vaccine, and 9% do not want it [15]. The population has not only had reluctance applying the COVID-19 vaccine, but also apply other vaccines. For example, Wang et al. [11] point out that only 58.6% of parents are willing to vaccinate their children routinely and, 47.3% are willing to vaccinate them against seasonal influenza.

While in China, 40% of men who have sex with men accepted COVID-19 vaccine [20]; 57.2% of people living with HIV and AIDS reported willingness to receive the COVID-19 vaccination [21], and 66% of adults with multiple sclerosis in the United Stated reported a willingness to obtain COVID-19 vaccine when available [22]. The meta-analysis study by Wang et al. [10] said that the COVID-19 vaccine was accepted by 81.65% of the adult population and 65.65% of HCWs. They estimated that the acceptance rate of the COVID-19 vaccine between populations was 73.31% and that the vaccination acceptance rate among the 20 countries and regions ranged from 43.38% to 94.31%; in this sampling, Mexico reported an acceptance rate of 76.25%, like Ecuador as countries in Latin America.

Studies on COVID-19 vaccination in different countries have explained the predictors of vaccination acceptance and hesitancy from the three theoretical levels of health behaviour, individual, interpersonal, and community [23], even though the majority has been at the personal level [8,[10], [11], [12],[14], [15], [16], [17],^19^,^20^,^24^]; cross-sectional studies in the adult and healthcare workers populations countries [10]. A large number of these studies explain the behaviour of the individual towards acceptance, uptake, willingness, intention, hesitancy and/or refusal of COVID-19 vaccines from sociodemographic characteristics as predictors of behaviour outside of theoretical framework and models of health behaviour [13,22,[25], [26], [27]]; while others analyse them from the social-ecological framework [28], Predisposing, Reinforcing and Enabling Constructs in Educational/Ecological Diagnosis and Evaluation (PRECEDE) model [20], health belief model (HBM) [10,13,14,18,29,30], and some use constructs of the theory of planned behaviour (TPB) [21,29].

The HBM and the TPB (extension of theory of reasoned action) are the most widely used theoretical models of behaviour to explain the determinants of health behaviour [31] and the vaccination intention [20] as the antecedent of behaviour. However, as far as we know, most of the studies on the predictors of COVID-19 vaccination acceptance or intention have been analysed from HBM, and few from TPB [21], but limited from an integrated model that combines the predictors of TPB and HBM [[32], [33], [34]]. In this study, in addition to these two widely used models in health behaviour, we added the model of goal-directed behaviour (MGB) framework, which contributes to explaining the individual's motivational, emotional, and habitual process to decision-making based on beliefs, emotions, motivations, rational actions and goals (predictors) to intend towards an action (vaccination behaviour).

So, this investigation aims to determine the predictors that have the greatest influence over COVID-19 vaccination intention for the population of 18 years or above in Mexico. This will be done through a comprehensive theoretical model comprising: TPB, HBM, and MGB. The investigation contributes towards identifying predictors through the three theories that best explain the intention of individuals towards emergency immunization like COVID-19 vaccination. In addition, this study provides information for decision-making on strategies to reduce uncertainty related to the lack of knowledge. It also contributes to the information generation on the predisposing factors as determinants of individual behaviour for intervention planners and promoters of behaviour changes towards vaccination for the PRECEDE- Policy, Regulatory, and Organisational Constructs in Educational and Environmental Development (PROCEED) model [31].

The following exploratory questions that will guide the investigation are posed.

● What variables from TPB, HBM, and MGB influence emerging vaccination intention like that for COVID-19?
● Which model best explains vaccination intention in an environment of doubt like the COVID-19 pandemic?

## 2 Models and theories for individual vaccination behaviour

Vaccination behaviour from HBM explores the individual beliefs, motives and reasons to accept or not COVID-19 vaccination; the model premise consists that there are beliefs that can predict future behaviour [29], including perceived susceptibility, perceived severity and perceived benefits, perceived barriers, and cues to action as predictors of vaccination intention [13,29,32,35]. These predictors related to the characteristics and understanding of the patient influence the beliefs of the subject and promote intention [33]. In Italy, Graffigna et al. [19], found that the susceptibility and severity influence disposition towards COVID-19 vaccination, and that these relationships are significant among middle-aged people. In contrast, according to Al-Metwali et al. [29], the HCWs who perceived the highest susceptibility and severity of COVID-19 were hesitant to receive the vaccine. Adults that perceived higher vaccine benefits, the effectiveness of the vaccine for protecting others and risks of COVID-19 transmission, as well as a sense belief that being vaccinated and following prevention measures increased their vaccination intention [12,14,20,26,29,36,37]. Although HBM is the most widely used to explain health behaviour from a cognitive perspective, it neglects the emotional predictors of individual behaviour, which TPB includes to explain behaviour towards an action [38].

The TPB is based on the individual's motivational factors as determinants of behaviour; it assumes that the best predictor of a behaviour is the intention [39], includes attitudes, subjective norms, and perceived control of behaviour to be the predictors of intention [40]. In Canada, upon analysing COVID-19 vaccination intention, it was found that both attitude (perception of the benefits of vaccination) and subjective norms (external opinions) are equally important predictors of vaccination intention [24]. Positive and negative attitudes, subjective norms (perceived support from others) and perceived behavioural control were positively associated with vaccination intention among people living with HIV and AIDS in China [21].

In turn, studies show that in the study of vaccination intention, TPB and HBM can be employed together [32,35]. In the analysis of COVID-19 vaccination, it is found that under these two theoretical models, 66% of vaccination intention is explained [32]. Meanwhile, in the analysis of H1N1 influenza vaccination, through both theoretical models, 60% of vaccination intention is explained [35].

However, there are other models that have not been tested in vaccination behaviour, but that have extended the understanding of the theory of planned behaviour in the field of health, including the motivational, emotional, and habitual processes in the analysis of behaviour, such as the MGB [41]. Goal-directed behaviour is involved in the analysis of well-being and health, as people pursue objectives related to health in their behaviour [42].

The MGB maintains the predictors of TPB, such as attitude defined as the perception of the benefits of vaccination, and subjective norms, which refer to external opinions. The perceived control of behaviour is also maintained, which is the perception of having control over being vaccinated. Added to this model is the desire of the individual, which indicates that through desire (considered to be the motivation to carry out behaviours to achieve specific goals), the individual will attempt to carry out the behaviour [40,41]. This is because behaviour is motivated by achievieng a goal, which is translated into the desire that the person seeks [41,42]. MGB adds other variables, such as anticipated emotions, as predictors of desire, and the frequency of previous behaviour as a predictor of behaviour intention. In this way, the model adds the emotional and habitual factors respectively to explain behaviour [41]. In the context of health, studies on MGB have been carried out, for example, the analysis of self-care intention for arterial hypertension, the intention of young people to carry out physical activity, and the intention of parents practices that foster the consumption of vegetables [[43], [44], [45]].

The study on the self-care intention for arterial hypertension revealed the causes for self-care intention were perceived control of behaviours, past behaviour, and desire. Meanwhile, attitude, subjective norms, perceived control of behaviour, and positive anticipated emotions explained the desire for self-regulation of arterial pressure [43]. The study on physical activity among young people showed that attitude, descriptive norms, perceived control of behaviour, and positive anticipated emotions, explained desire, whilst desire explained the intention to carry out physical activity [44]. Hingle et al. [45] explained the factors that motivate parents to have practices that foster the consumption of vegetables which were attitudes, positive and negative emotions, subjective norms, and perceived control of behaviour.

From a theoretical model, it is possible to identify the factors that best predict vaccination intention. For example, TPB allows the identification of whether the factors for getting vaccinated are only based on the person or external people; MGB allows a better understanding of motivations for getting vaccinated, and HBM considers the risks and benefits associated with vaccination. In this way, the design of vaccination strategies will be possible, and as such, so too will the reduction of infections or hospitalisations be possible. Thus, in this study, vaccination intention is analysed by integrating the three main theories to explain COVID-19 vaccination intention: the TPB, HBM and MGB. This is to determine the model that best explains vaccination intention for the population of 18 years and older in Mexico in the face of a health emergency like COVID-19.

## 3 Method

**Figure 1:**
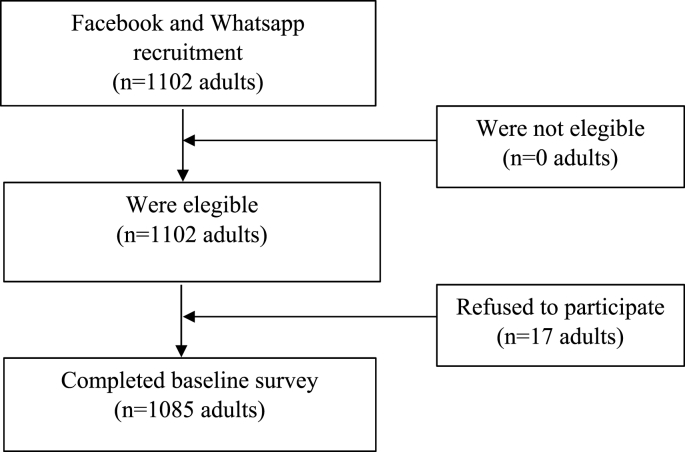
Flowchart of data collection process.

An exploratory, transversal study with a quantitative approach was carried out. The structured questionnaire was applied in the first trimester of the year 2021 through Google forms in social media groups on Facebook and Whatsapp. The study was conducted according to the guidelines of the Declaration of Helsinki and approved by the Secretaria de Investigación y Posgrado of the Instituto Politécnico Nacional with the reference number SIP20211418. The study complies with all regulations. The consent of the participants, who were above the age of 18, was obtained to carry out the study. As other studies such as Zhang et al. [20], a flowchart of the collection data was shown in Fig. 1. The questionnaire was applied in April 2021, when the second stage of vaccine applications was being carried out in Mexico (adults above the age of 60) [46]. The available vaccines in this vaccination period were Pfizer-BioNTech, AstraZeneca, Sinovac, and SputnikV [47].

The size of the sample was 1085, which covered the minimum requirement for data treatment through partial least square equation modelling, obtained through statistical power analysis [48]. Expansion factors were not considered, so the sample is representative of its sociodemographic characteristics. In statistical power analysis, the effect size was small to guarantee a conservative approximation. The number of predictors was 6, the significance level was 0.1, and the statistical power was 0.8 [[48], [49], [50]]. Considering said values, the minimum sample size required was 953 [50]. The data analysis was carried out through partial least square structural equation modelling (PLS-SEM) using the SmartPLS 3.3.7 software [51]. Richter [52] argues that PLS-SEM offers better results for prediction-oriented models than other types of analysis. Since the study analyses a predictive individual behaviour model, this statistical technique was selected. This software can be used when the structural model is complex with many constrictions and relationships [53], as is the case for the models in this investigation.

Of the respondents, 35.9% were men, and 64.1% were women. 43.6% of the sample were between 18 and 30 years old, whilst 20.7% were between 31 and 40 years old. 20.5% of respondents were between 41 and 50 years old, whilst 8.7% were between 51 and 60 years old. The rest were more than 61 years old. 55.8% of respondents had a bachelor's degree, 17.9% had a master's degree, 14% had a *bachillerato*, 10.6% had a doctorate degree, and the rest had less than a high-school education. The civil status of 49.4% of the sample was single, whilst 33.2% were married (Table 1).

**Table 1:** Desciptive data.

Descriptive Data		Frequency	Frequency (%)
Gender	Female	695	64.1
	Male	390	35.9
Age	18 years–30 years	473	43.6
	31 years–40 years	225	20.7
	41 years–50 years	222	20.5
	51 years–60 years	94	8.7
	61 years or more	71	6.5
Level of studies	Primary Incomplete	2	0.2
	Primary	5	0.5
	Secondary	12	1.1
	*Bachillerato*	152	14
	Bachelor's Degree	605	55.8
	Master's Degree	194	17.9
	Doctorate Degree	115	10.6
Civil status	Married	360	33.2
	Single	536	49.4
	Consensual Union	122	11.2
	Separated	25	2.3
	Divorced	26	2.4
	Widowed	13	1.2
	Other	3	0.3

### 3.1 Measures

The TPB constructs include attitudes, precautionary norms, descriptive norms, perceived control of behaviour, and vaccination intention. The attitude construct was defined as the favourable evaluation of vaccination. The scale was adapted from Myers and Goodwin [35] and it was composed of two items. Subjective norms were defined operationally as the perceived grade of approval for a behaviour (vaccination). The scale was adapted from Myers and Goodwin [35] and it was composed of three items. The perceived control of behaviour was adopted by Guidry et al. [32], and was defined as individual control over receiving the vaccine (three items). Vaccination intention was defined as the disposition towards getting vaccinated in the considering similar pandemics. It was adapted in two items from the scales by Guidry et al. [32], Myers and Goodwin [35] and Wang [54].

The HBM constructs were perceived susceptibility, perceived severity, and perceived benefits, which were adapted from Myers and Goodwin [35]. Perceived susceptibility was defined operationally as the individual perception of contracting the disease soon. Perceived severity was defined as the perceived risk of disease infection. Perceived benefits were defined as the perceived reduction of the risks of contagion or complications due to the disease through getting vaccinated. Each of the HBM constructs was composed of three items.

The MGB constructs included those from TPB, as well as desire, frequency of past behaviour, and positive and negative anticipated emotions. Desire was defined as the level of zest towards getting vaccinated to care for one's personal, family, and social health [41]. It was composed of three items. Frequency of past behaviour was defined as past vaccination behaviour [35], and was composed for three items. The construct of positive anticipated emotions was defined as positive individual feelings like safety, pride, and satisfaction due to achieving goals related to taking care of one's health. It was adapted in six items from Perugini and Bagozzi [41]. The construct of negative anticipated emotions was defined as negative individual feelings such as anger, sadness, frustration, and discomfort due to failing to realise goals related to caring for one's health [41]. It was composed of seven items.

The scale for all the constructs of the study was a 5-point Likert scale: 1 - totally disagree, to 5 - totally agree, with the except for the desire construct, which used: 1 - very weak, to 5 - very strong. All the constructs used in the study are shown in Table 2.

**Table 2:** Constructs in the study.

Construct	Item	
Attitude	8ACT	For me, getting vaccinated is beneficial in preventing contagious diseases.
	9ACT	For me, getting vaccinated is positive in preventing serious diseases.
Subjective norms	10NCA	The majority of the people who are important to me believe that I should get vaccinated against COVID-19 or other illnesses.
	11NCA	Some people who influence my opinion believe that I should get vaccinated against COVID-19 or other illnesses.
	12NCA	My close friends and family believe that getting vaccinated is good.
Perceived behavioural control	16CPE	It's up to me to receive the COVID-19 vaccine when it's available.
	17CPE	It's up to me to receive a vaccine against other illnesses.
Intention	26IVS	I will definitely try to get vaccinated against diseases similar to this pandemic in the future.
	28IVS	I will continue to be interested in getting vaccinated against diseases that can cause damage to my health in the future
Desire	22DES	My desire to care for my health by getting vaccinated against COVID-19 is
	23DES	My desire to care for the health of people that are important to me by getting vaccinated against COVID-19 is
	24DES	My desire to care for the health of my community by getting vaccinated against COVID-19 is
Frequency of past behaviour	19FCP	Last year I received the vaccine against influenza.
	20FCP	Years ago, I received the vaccine against influenza.
	21FCP	My immunization schedule is complete.
Positive anticipated emotions	31EAP	If I manage to achieve my goal of caring for my health through getting vaccinated against COVID-19, I will feel delighted.
	34EAP	If I manage to achieve my goal of caring for my health through getting vaccinated against COVID-19, I will feel satisfied.
	36EAP	If I manage to achieve my goal of caring for my health through getting vaccinated against COVID-19, I will feel safe.
	42EAP	If I manage to achieve my goal of caring for the health of the people who are important to me through getting vaccinated against COVID-19, I will feel proud.
	44EAP	If I manage to achieve my goal of caring for the health of my community through getting vaccinated against COVID-19, I will feel proud.
	50EAP	If I manage to achieve my goal of caring for the health of my community through getting vaccinated against COVID-19, I will feel safe.
Negative anticipated emotions	51EAN	If I don't manage to achieve my goal of caring for my health through getting vaccinated against COVID-19, I will feel angry.
	56EAN	If I don't manage to achieve my goal of caring for my health through getting vaccinated against COVID-19, I will feel disappointed.
	60EAN	If I don't manage to achieve my goal of caring for my health through getting vaccinated against COVID-19, I will feel tense.
	61EAN	If I don't manage to achieve my goal of caring for the health of the people who are important to me through getting vaccinated against COVID-19, I will feel angry.
	65EAN	If I don't manage to achieve my goal of caring for the health of the people who are important to me through getting vaccinated against COVID-19, I will feel sad.
	72EAN	If I don't manage to achieve my goal of caring for the health of my community through getting vaccinated against COVID-19, I will feel frustrated.
	79EAN	If I manage to achieve my goal of caring for the health of my community through getting vaccinated against COVID-19, I will feel uncomfortable.
Perceived susceptibility	81SPE	My probability of contracting COVID-19 in the next few months is high.
	82SPE	I'm worried about contracting COVID-19 in the near future.
	83SPE	Contracting COVID-19 is currently a possibility for me.
Perceived benefits	87BEN	Vaccination reduces my worries about contracting COVID-19.
	88BEN	Vaccination reduces the possibility of me contracting COVID-19 or its complications.
	89BEN	If I get vaccinated against COVID-19, I will reduce the probability of getting hospitalised by COVID-19.
Perceived severity	84SEV	The complications of COVID-19 are serious.
	85SEV	I will be very frail if I contract COVID-19.
	86SEV	I'm afraid of contracting COVID-19.

Source: constructs adapted from Guidry et al. (2021), Myers and Goodwin (2011), Perugini & Bagozzi (2001), Wang (2020).

### 3.2 Procedures a data analysis

The data analysis was carried out through PLS-SEM. The evaluation of the data was integrated by evaluating the measurement model, the structural model, and the adjustment of the global model [48,55]. Through PLS-SEM, the predictions of the model are optimised without assuming a type of distribution in which the variables are standardised [56], and it is written as:

ŋ=Bŋ+ɽɛ+ƈ

According to de la Concha [56], the endogenous latent variables are denoted with the ŋ and the exogenous latent variables with the ɛ, and the error with the ƈ. The coefficients of these variables are *B* and ɽ.

The evaluation of the measurement model included the evaluation of: the reliability of the indicators (factor loadings), the reliability of the construct (Cronbach's alpha, Dijkstra and Henseler's rho, compounded reliability), the convergent validity (average variance explained), and the discriminant validity (Fornell-Larcker criterion, cross-loading analysis, Heterotrait-Monotrait Ratio) [48,57]. In the case of the structural model, the collinearity was evaluated (variance inflation factor). The predictive relevance was determined (determination coefficient R^2^ and Stone-Geisser's Q^2^ value), as was the significance and relevance of the path coefficients [57,58]. The evaluation of the adjustment of the model was carried out through the approximate adjustment of the model through standardised root mean square residual (SRMR) [59]. The consistent PLS algorithm was used, blindfolding with a value D = 6, and bootstrapping with 5000 subsamples in the data analysis [60,61].

## 4 Results

In the evaluation of the measurement model, the reliability values of the indicators (loads) higher than 0.5 were kept, as said indicators can contribute to the content [62]. The reliability values of the construct are higher than 0.707, which means that the indicators reliably represent the construct [48]. In the evaluation of the convergent validity, as shown in Table 3, values higher than 0.5 of the average variance extracted (AVE) were valid, by Hair et al. [63].

**Table 3:** Reliability and convergent validity.

Construct	Item	Load	AVE	ρA	CR	α
Attitude	8ACT	0.986	0.911	0.955	0.953	0.952
	9ACT	0.922				
Subjective norms	10NCA	0.942	0.761	0.909	0.905	0.904
	11NCA	0.838				
	12NCA	0.832				
Perceived behavioural control	16CPE	0.891	0.831	0.909	0.908	0.907
	17CPE	0.932				
Intention	26IVS	0.962	0.879	0.937	0.936	0.935
	28IVS	0.913				
Desire	22DES	0.918	0.761	0.907	0.905	0.906
	23DES	0.861				
	24DES	0.837				
Frequency of past behaviour	19FCP	0.744	0.609	0.827	0.823	0.824
	20FCP	0.753				
	21FCP	0.841				
Positive anticipated emotions	31EAP	0.800	0.687	0.932	0.929	0.930
	34EAP	0.900				
	36EAP	0.887				
	42EAP	0.751				
	44EAP	0.771				
	50EAP	0.854				
Negative anticipated emotions	51EAN	0.905	0.691	0.942	0.940	0.940
	56EAN	0.823				
	60EAN	0.800				
	61EAN	0.825				
	65EAN	0.839				
	72EAN	0.893				
	79EAN	0.718				
Perceived susceptibility	81SPE	0.561	0.559	0.851	0.782	0.786
	82SPE	0.980				
	83SPE	0.634				
Perceived benefits	87BEN	0.838	0.795	0.923	0.921	0.921
	88BEN	0.894				
	89BEN	0.941				
Perceived severity	84SEV	0.886	0.516	0.795	0.753	0.760
	85SEV	0.521				
	86SEV	0.700				

AVE-variance extracted from the mean, ρA- Dijkstra and Henseler's rho, α- Cronbach's alpha, CR-composite reliability.

The cross-loading analysis allowed the evaluation of the discriminant validity, as in said analysis, it was observed that the items had a greater in the construct shown in Table 4 [61]. The Fornell-Larcker criterion also allowed the evaluation of the discriminant validity by verifying that the square root of each constructs's AVE value was greater than its correlations with other constructs, as ascertained in Table 5 [57]. Meanwhile, the Heterotrait-Monotrait Ratio values allowed the detection of discriminant validity problems if they were greater than 0.85 [63], all the values were less than this value (see Table 6).

**Table 4:** Cross-loading analysis.

Construct	Item	Cross loadings analysis										
		1	2	3	4	5	6	7	8	9	10	11
1.Attitude	8ACT	**0.986**	0.735	0.569	0.562	0.363	0.363	0.426	0.256	0.269	0.333	0.288
	9ACT	**0.922**	0.705	0.552	0.533	0.339	0.365	0.392	0.253	0.241	0.322	0.265
2.Subjective norms	10NCA	0.709	**0.942**	0.626	0.622	0.453	0.436	0.502	0.301	0.322	0.385	0.352
	11NCA	0.589	**0.838**	0.539	0.529	0.403	0.418	0.450	0.289	0.270	0.339	0.322
	12NCA	0.673	**0.832**	0.600	0.579	0.400	0.407	0.449	0.287	0.265	0.350	0.306
3.Perceived behavioural control	16CPE	0.548	0.624	**0.891**	0.455	0.274	0.386	0.324	0.206	0.160	0.231	0.247
	17CPE	0.524	0.608	**0.932**	0.477	0.286	0.416	0.308	0.203	0.162	0.202	0.202
4.Intention	26IVS	0.532	0.627	0.461	**0.962**	0.612	0.397	0.672	0.363	0.394	0.515	0.470
	28IVS	0.544	0.614	0.500	**0.913**	0.579	0.405	0.653	0.331	0.367	0.481	0.419
5.Desire	22DES	0.339	0.429	0.265	0.589	**0.918**	0.292	0.600	0.349	0.324	0.490	0.380
	23DES	0.335	0.424	0.286	0.551	**0.861**	0.290	0.557	0.325	0.320	0.445	0.422
	24DES	0.287	0.405	0.254	0.521	**0.837**	0.274	0.564	0.301	0.285	0.451	0.380
6.Frequency of past behaviour	19FCP	0.244	0.345	0.289	0.318	0.244	**0.744**	0.302	0.185	0.162	0.222	0.169
	20FCP	0.295	0.354	0.323	0.322	0.263	**0.753**	0.275	0.174	0.104	0.199	0.138
	21FCP	0.349	0.425	0.413	0.360	0.259	**0.841**	0.283	0.191	0.169	0.212	0.152
7.Positive anticipated emotions	31EAP	0.346	0.431	0.254	0.572	0.526	0.309	**0.800**	0.422	0.324	0.522	0.436
	34EAP	0.375	0.470	0.318	0.653	0.592	0.348	**0.900**	0.413	0.377	0.594	0.465
	36EAP	0.390	0.491	0.328	0.643	0.583	0.310	**0.887**	0.413	0.357	0.568	0.430
	42EAP	0.335	0.419	0.273	0.519	0.494	0.254	**0.751**	0.398	0.251	0.472	0.394
	44EAP	0.333	0.408	0.259	0.537	0.508	0.287	**0.771**	0.394	0.312	0.520	0.417
	50EAP	0.352	0.444	0.283	0.579	0.562	0.310	**0.854**	0.360	0.293	0.531	0.394
8.Negative anticipated emotions	51EAN	0.236	0.284	0.217	0.323	0.338	0.212	0.413	**0.905**	0.316	0.412	0.369
	56EAN	0.236	0.287	0.189	0.317	0.307	0.186	0.377	**0.823**	0.313	0.390	0.350
	60EAN	0.206	0.252	0.179	0.298	0.299	0.175	0.360	**0.800**	0.361	0.390	0.363
	61EAN	0.249	0.303	0.218	0.335	0.308	0.205	0.417	**0.825**	0.339	0.406	0.385
	65EAN	0.236	0.300	0.199	0.322	0.313	0.212	0.430	**0.839**	0.334	0.372	0.381
	72EAN	0.202	0.274	0.147	0.293	0.333	0.179	0.435	**0.893**	0.335	0.401	0.386
	79EAN	0.184	0.249	0.156	0.266	0.268	0.201	0.366	**0.718**	0.342	0.358	0.375
9.Perceived susceptibility	81SPE	0.156	0.172	0.054	0.228	0.178	0.082	0.187	0.206	**0.561**	0.206	0.380
	82SPE	0.278	0.334	0.208	0.398	0.372	0.209	0.418	0.411	**0.980**	0.445	0.660
	83SPE	0.140	0.201	0.096	0.258	0.207	0.097	0.212	0.243	**0.634**	0.283	0.455
10.Perceived benefits	87BEN	0.283	0.343	0.219	0.445	0.458	0.221	0.555	0.432	0.388	**0.838**	0.476
	88BEN	0.302	0.362	0.221	0.475	0.456	0.246	0.575	0.419	0.368	**0.894**	0.503
	89BEN	0.331	0.394	0.198	0.500	0.503	0.253	0.598	0.408	0.409	**0.941**	0.536
11.Perceived severity	84SEV	0.272	0.332	0.244	0.420	0.386	0.166	0.433	0.298	0.461	0.463	**0.886**
	85SEV	0.140	0.203	0.113	0.247	0.222	0.074	0.266	0.248	0.478	0.318	**0.521**
	86SEV	0.192	0.257	0.149	0.332	0.343	0.168	0.381	0.427	0.573	0.429	**0.700**

The highest loads are in bold.

**Table 5:** Fornell-Larcker criterion.

Construct	Fornell-Larcker criterion										
	1	2	3	4	5	6	7	8	9	10	11
1.Attitude	**0.954**										
2.Subjective norms	0.755	**0.872**									
3.Perceived behavioural control	0.587	0.675	**0.912**								
4.Intention	0.574	0.662	0.511	**0.937**							
5.Desire	0.368	0.481	0.307	0.635	**0.872**						
6.Frequency of past behaviour	0.381	0.482	0.441	0.428	0.327	**0.781**					
7.Positive anticipated emotions	0.429	0.536	0.346	0.707	0.658	0.367	**0.829**				
8.Negative anticipated emotions	0.267	0.335	0.224	0.371	0.373	0.235	0.482	**0.831**			
9.Perceived susceptibility	0.267	0.328	0.176	0.406	0.355	0.186	0.386	0.401	**0.748**		
10.Perceived benefits	0.343	0.411	0.238	0.532	0.530	0.270	0.646	0.469	0.435	**0.892**	
11.Perceived severity	0.290	0.375	0.245	0.475	0.451	0.196	0.510	0.448	0.685	0.567	**0.718**

The square root of the AVE is on the diagonal (bold).

**Table 6:** Heterotrait-monotrait ratio.

Construct	Heterotrait-Monotrait Ratio										
	1	2	3	4	5	6	7	8	9	10	11
1.Attitude											
2.Subjective norms	0.756										
3.Perceived behavioural control	0.589	0.677									
4.Intention	0.575	0.663	0.513								
5.Desire	0.367	0.481	0.307	0.634							
6.Frequency of past behaviour	0.380	0.481	0.437	0.428	0.327						
7.Positive anticipated emotions	0.429	0.536	0.345	0.705	0.656	0.366					
8.Negative anticipated emotions	0.267	0.336	0.224	0.370	0.371	0.235	0.481				
9.Perceived susceptibility	0.258	0.317	0.161	0.397	0.340	0.174	0.365	0.388			
10.Perceived benefits	0.343	0.411	0.239	0.531	0.529	0.270	0.645	0.470	0.420		
11.Perceived severity	0.281	0.369	0.236	0.465	0.442	0.190	0.502	0.453	0.693	0.563	

In the evaluation of the structural model, the collinearity should be evaluated before the structural relations. This is to corroborate that there is no bias in the regressions, and as a criterion, that the variance inflation factor (VIF) values should be less than 5 [63]. The VIF values of the relationships of all the models are less than this value, as shown in Table 7. After evaluating the collinearity, the size of the effect was evaluated [59]. The biggest effect size of the relationships was that of positive anticipated emotions in the intention of model 3 (f^2^ = 0.180), which was moderate due to being greater than 0.15 and smaller than 0.35 [64]. The determination coefficient (R^2^) represents the degrees of explained variance [48], the greater the value, the better explained the studied phenomenon. The R^2^ of the endogenous intention construct were compared to determine the model that best explains the phenomenon. The R^2^ values that are bigger than or equal to 0.25 are weak, the values bigger than or equal to 0.5 are moderate, and the values bigger than or equal to 0.75 are substantial [65]. The Q^2^ values show the predictive accuracy values of the model: values greater than 0.25 and lower than 0.50 indicate moderate predictive accuracy, whilst values greater than 0.50 represent great predictive accuracy [63] The models that include the variables for MGB had a great predictive accuracy (Models 3 and 5), whilst the others had a moderate predictive accuracy (Models 1, 2, and 4). The path coefficients are the standardised regression coefficients [48], and in this sense, they show the effect of an exogenous construct on an endogenous construct. The adjustment of the models was carried out through the SRMR value. It was verified that this value was less than 0.08 to guarantee an acceptable adjustment [48,59]. All the SRMR values of the models were less than 0.08, and as such, all the proposed models had a good adjustment (Table 7).

**Table 7:** Evaluation of the structural model and global model.

Model	Exogenous construct	Endogenous construct	β	t	p	f^2^	VIF
1.TPB SRMR = 0.019	Attitude	Intention **R**^**2**^=**0.455 Q**^**2**^=**0.378**	0.156	3.072	0.002	0.019	2.389
	Subjective norms		0.478	8.701	0.000	0.146	2.876
	Perceived behavioural control		0.098	1.902	0.057	0.009	1.887
2.HBM SRMR = 0.056	Perceived susceptibility	Intention **R**^**2**^=**0.292 Q**^**2**^=**0.271**	0.133	4.442	0.000	0.017	1.501
	Perceived benefits		0.367	9.124	0.000	0.142	1.341
	Perceived severity		0.157	4.128	0.000	0.021	1.672
3.MGB SRMR = 0.025	Attitude	Intention **R**^**2**^=**0.656 Q**^**2**^=**0.552**	0.128	2.894	0.004	0.020	2.396
	Subjective norms		0.172	3.051	0.002	0.025	3.405
	Perceived behavioural control		0.109	3.071	0.002	0.018	1.956
	Desire		0.221	4.181	0.000	0.077	1.851
	Positive anticipated emotions		0.368	6.387	0.000	0.180	2.194
	Negative anticipated emotions		−0.015	0.593	0.553	0.001	1.323
	Frequency of past behaviour		0.045	1.943	0.052	0.004	1.383
4.TPB + HBM SRMR = 0.045	Attitude	Intention **R**^**2**^=**0.552 Q**^**2**^=**0.464**	0.127	2.727	0.006	0.015	2.405
	Subjective norms		0.322	5.586	0.000	0.074	3.130
	Perceived behavioural control		0.126	2.902	0.004	0.019	1.909
	Perceived susceptibility		0.070	1.786	0.074	0.006	1.922
	Perceived benefits		0.236	5.271	0.000	0.078	1.597
	Perceived severity		0.104	2.074	0.038	0.011	2.297
5. TPB + HBM + MGB SRMR = 0.039	Attitude	Intention **R**^**2**^=**0.663 Q**^**2**^=**0.560**	0.124	2.794	0.005	0.019	2.407
	Subjective norms		0.157	2.785	0.005	0.021	3.435
	Perceived behavioural control		0.116	3.292	0.001	0.020	1.981
	Desire		0.202	3.953	0.000	0.064	1.918
	Positive anticipated emotions		0.338	5.907	0.000	0.134	2.560
	Negative anticipated emotions		−0.048	1.782	0.075	0.005	1.467
	Frequency of past behaviour		0.047	2.016	0.044	0.005	1.391
	Perceived susceptibility		0.073	2.175	0.030	0.008	1.955
	Perceived benefits		0.032	0.819	0.413	0.001	2.072
	Perceived severity		0.032	0.726	0.468	0.001	2.392

β-path coefficient, t-t value, p-p value, f^2^-effect size, VIF- variance inflation factor.

To explain vaccination intention, 5 models were posed which include the variables from TBP, HBM, and MGB, through simple analysis and combined from models 1–5 (Table 7). It was found that in model 1, which includes the variables from TPB, the subjective norms best explain vaccination intention (β = 0.478, p = 0.000), followed by attitude (β = 0.156, p = 0.002). The explanation of this model was 45.5% (R^2^ = 0.455, p = 0.000). For model 2 of the HBM, the explanation was weak but significant (R^2^ = 0.292, p = 0.000). In this model, the perceived benefits were the greatest predictor for vaccination intention (β = 0.367, p = 0.000), allowed by the perceived severity (β = 0.157, p = 0.000) and perceived susceptibility (β = 0.133, p = 0.000). In model 3, which analyses the MGB variables, the positive anticipated emotions were the variable that had the greatest influence on intention (β = 0.368, p = 0.000), followed by desire (β = 0.221, p = 0.000), subjective norms (β = 0.172, p = 0.002), attitude (β = 0.368, p = 0.000), and perceived behavioural control (β = 0.368, p = 0.000). The explanation of model 3 was moderate and significant (R^2^ = 0.656, p = 0.000).

Through the multiple analysis of models 1–3, it was found that upon including the variables for TPB and HBM in the analysis, the main predictors for vaccination intention were subjective norms (β = 0.322, p = 0.000) and perceived benefits (β = 0.236, p = 0.000), followed by perceived behavioural control (β = 0.106, p = 0.004), attitude (β = 0.127, p = 0.006), and perceived severity (β = 0.104, p = 0.038). Perceived susceptibility was not significant. The explained variance of the model was 55.2% (R^2^ = 0.552, p = 0.000). In model 5, variables from the three theories (TPB, HBM, and MGB) were included, and it was found that the two most influential predictors for vaccination intention were positive anticipated emotion (β = 0.555, p = 0.000) and desire (β = 0.555, p = 0.000). Subjective norms (β = 0.157, p = 0.005), attitude (β = 0.124, p = 0.005), perceived behavioural control (β = 0.116, p = 0.001), perceived susceptibility (β = 0.073, p = 0.030), and frequency of past behaviour (β = 0.047, p = 0.044) were also positive and significant. The explanation of model 5 was 66.3% and was the model that explained vaccination intention in the greatest proportion (R^2^ = 0.663, p = 0.000).

**Figure 2:**
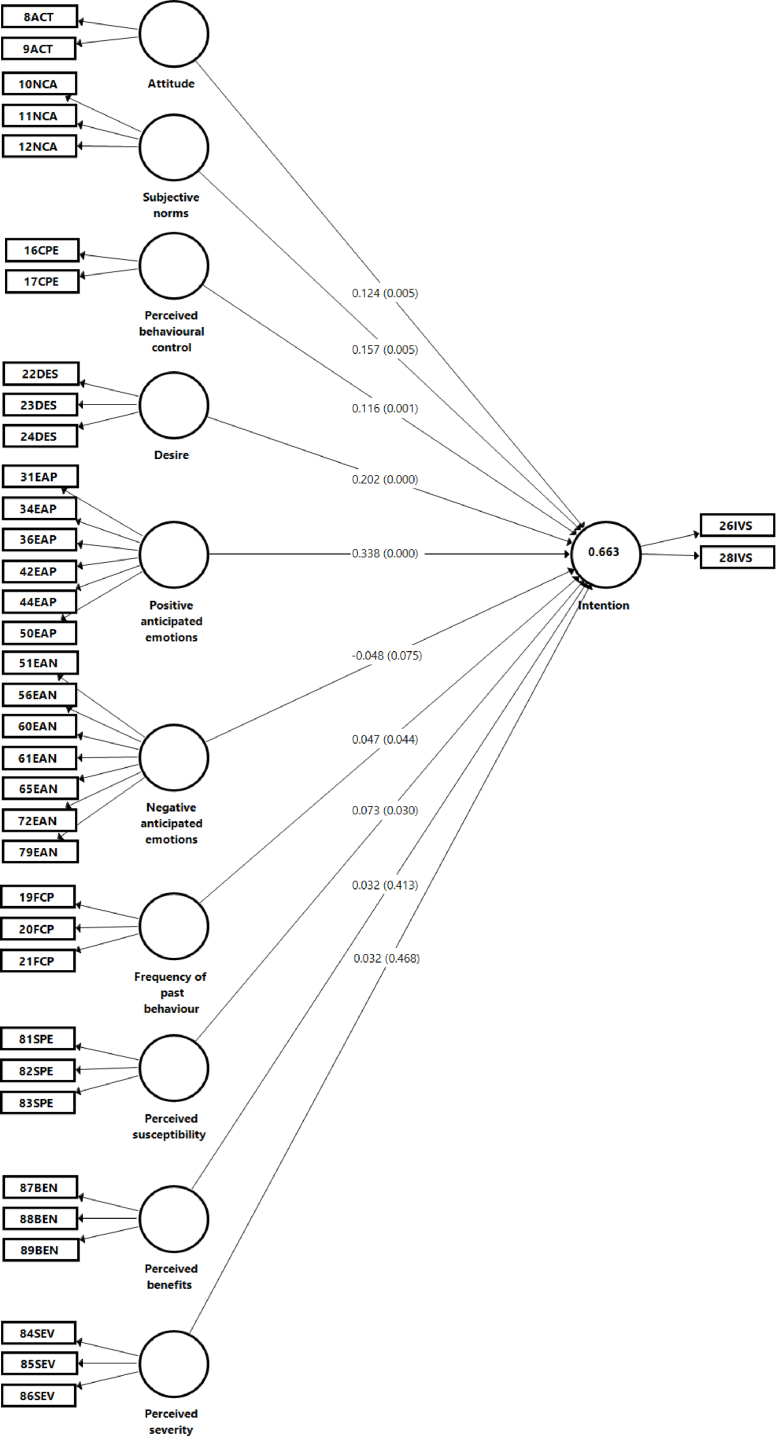
Model 5, integrating TPB, HBM, and MGB. R^2^ values are in the endogenous constructs, path coefficients are in the arrows, and p-values are in parentheses.

The variables of subjective norms from TPB, perceived benefits from HBM, and positive anticipated emotions from MGB had the greatest influence on each theory in explaining vaccination intention (Models 1–3). When combined, in model 5, positive anticipated emotions, desire, subjective norms, attitude, and perceived behavioural control were the greatest predictors of intention, whilst frequency of past behaviour and perceived susceptibility were to a lower degree (Fig. 2).

## 5 Discussion

The results of this investigation show that, from the theoretical framework of the TPB, the HBM, and the MGB, it is possible to exlplain the predictors that determine whether individuals will have the intention to get vaccinated. Their contributes to explaining the willingness and hesitancy among the population towards vaccination, and that it is key in both economic recovery and the reduction of sanitary crises like that generated by the COVID-19 pandemic. According to the data from the applied survey, 66.5% of adults in Mexico intend to get the COVID-19 vaccine when it becomes available according to phase-by-age ranges programmed in México. This vaccination intention rate coincides with the 62.3% national vaccination rate in Mexico [66], 12.9% of adult participants have a moderate intention, and 4.3% has little intention to get COVID-19 vaccination. Whilst 10% of the participants were unsure of their intention, they decided not to respond to the question, and 6.3% refused the intention to get the vaccine. The intention estimation rate of Mexicans was 83.7%. This acceptance rate is similar to the estimation rate determined by Wang et al. [10] on the intention to vaccinate the adult population in general (81.65%) from 20 countries where the acceptability of the COVID-19 vaccine was studied. This vaccination intention rate is slightly higher in 70% of adults reported by Reiter et al. [37] in the United States; this rate is similar to the 63.1% of adult population willingness in the meta-analysis of Limbu et al. [13]; the rate similar to 85% of Ecuador population, who is willing to pay to receive the COVID-19 vaccine, and the parental willingness for children vaccination (58.6%) reported by Wang et al., [11].

Analysing both the HBM and TPB, the results show that the benefits and perceived benefits of HBM, and the subjective norms and attitude of TPB, maintain their positive influence and significance for vaccination intention. However, the perceived susceptibility was not significant (Model 4). The individuals have the intention to get COVID-19 vaccine by the influence and protection of others, so they evaluate COVID-19 vaccination positively. They accept vaccines to protect themselves and others from infection. This model better explains the intention to vaccinate than when only each theoretical model is analysed separately (Models 1 and 2).

The MGB results signal that positive anticipated emotions, desire, and subjective norms strongly effect on vaccination intention (Model 3). For study participants in Mexico, the opinion of others on their behaviour, the feeling of satisfaction, safety, pride, and emotion generates the desire of the individual to guarantee care for their personal and social health through getting vaccinated, all positively influence in the intetion to get COVID-19 vaccine (Model 3). These results differ from those of other studies where other behaviours related to health are analysed, but that do not severely impact the health of the individual, such as self-care for arterial hypertension, physical activity, and healthy food, where attitude and perceived behavioural control influence intention [[43], [44], [45]]. The severity of the effects explains said differences, given that when severity increases, the individual can put volitive processes to one side and act more on emotions and external opinions.

Upon incorporating the HBM to the MGB, which includes the variables from TPB (model 5), positive anticipated emotions and desire are the most significant predictors in explaining intention. They are followed by subjective norms, attitudes, and perceived behavioural control. Frequency of past behaviour and perceived susceptibility are also added to the model, and although they have a lower coefficient, they are positive. The severity and the perceived benefits were not significant in the model. The predictors as susceptibility perceived to contracting COVID-19 disease, feelings of emotion and pride in achieving vaccination, the desire to take care of their health and that of others, autonomy decision to obtain the vaccine, and vaccination of seasonal influenza histories and other routine vaccinations (frequency of past behaviour) influence the intention to get COVID-19 vaccine among adult participants. In this integrated model, the motivational and emotional process postulated by Perugini y Bagozzi [41] explains that the intention towards behaviour changes by individual desire, positive anticipated emotion and subjective norms. However, add to the model the predictor of frequency of past behaviour like a routine vaccination action.

The predictors of vaccination intention identified among participants in Mexico coincide with studies from different contexts. For example, the importance of seasonal influenza vaccination history, which influence to accept the COVID-19 vaccine [10,11,13], the concern for health-care of others increases the COVID-19 vaccine willingness of general population [10,28,30], and perceived susceptibility to COVID-19 infection influence in acceptance of vaccine [11,37,67].

The field work of this study, as well as the collection of data, took place during the COVID-19 pandemic, whilst in Mexico the first vaccines against said disease were being applied in urban entities within the country (medical personnel and adults older than 60). In this period (first trimester 2021), there existed an asymmetry of information regarding the secondary effects and origin of the applied vaccines, which generated uncertainty in the general population. As such, one of the limitations of this study was its scope, due to the technique for information collection (web), which prevented the survey from taking place where access to the internet is limited. Another limitation was that the sample was comprised volunteers who accepted the invitation to participate through digital contacts.

Another limitation of the research was the measurement of the intention of the vaccination behaviour that does not necessarily translate into the current vaccine behaviour, in addition, that only the predictors of an individual health behaviour were evaluated, so the results do not reflect the current behaviour. In turn, an adult population was analysed that does not reflect the entire Mexican population, and the fact that there were no expansion factors, with which it is not possible to generalise. The sample is representative of its characteristics and the findings should be taken with caution because individual behaviour can change over time with context. It is recommended that future research perform a longitudinal study to assess individual behaviour over time and thereby reduce bias. However, the model can be used in developing vaccination promotion strategies, even as a theoretical basis for future research that analyses PRECEDE model for designing intervention programs.

The study can be a useful theoretical perspective for understanding similar behavioural intentions related to health risks, not only for vaccination intention. The results are also useful for decision makers in public health for the design of strategies that incentivise vaccination, given that it has been seen that there is still hesitancy towards vaccination in all sectors of the population, and that it is essential for health control.

## 6 Conclusions

Framework integrated by HBM, TPB and MBG contribute to better explaining the intention of vaccination from the individual's cognitive, emotional, and motivational perspectives like theories of health behaviour determinants as to COVID-19 diseases. Our research demonstrates that vaccination intention is influenced by positive anticipated emotions, desire, subjective norms, attitude, perceived control of behaviour, and perceived susceptibility and frequency of past behaviour. This integrated model can be a theoretical framework used to understand the intention of behaviours in an uncertain and contingency environment such as the COVID-19 pandemic, where health authorities slowly diffused the information; it generated deferred perceptions among the adult populations to receive whether to COVID-19 vaccine, and so that achieve a higher vaccination rate. The information generated can be helpful in the design of strategies that promote vaccination and the interventions planning to process individual and community health behavioural changes.

## Ethics statement

The study was conducted according to the guidelines of the Declaration of Helsinki and approved by the Secretaria de Investigación y Posgrado of the Instituto Politécnico Nacional with the reference number SIP20211418. The consent of the participants was obtained to carry out the study.

## Data availability statement

The data that support the findings of this study are available from the corresponding author.

## Declaration of competing interest

The authors declare that they have no known competing financial interests or personal relationships that could have appeared to influence the work reported in this paper.
